# Capacity measurement of a recurrent inhibitory neural network

**DOI:** 10.1186/1471-2202-12-S1-P196

**Published:** 2011-07-18

**Authors:** Chun-Wei Yuan, Christian Leibold

**Affiliations:** 1 Division of Neurobiology, Department of Biology II, Ludwig Maximilians Universität, 82152 Martinsried, Germany

## 

Inhibitory neurons are considered to play a central role as rhythm generator and in shaping feed-forward receptive fields. While much attention has been paid to such effects on excitatory neurons, little is done to study these inhibitory neurons' ability to directly process information. Here we present a model that investigates the computational capacity of a recurrent inhibitory neural network.

Our work focuses on quantifying the performance of a recurrent network of inhibitory integrate-and-fire neurons in canonical classification tasks. The model begins with parallel independent excitatory Poisson inputs connected to the recurrent network. Then, the network output is feed-forwardly directed to a read-out linear classifier. An identical network, but with zero synaptic connectivity, is set up for benchmarking. The analysis is then conducted by comparing the capacities of both setups, at 95% accuracy, as a function of parameters such as inhibitory weight, network size, etc.

It is found that, in general, neurons with faster time constants provide better computational power. Furthermore, there is an optimum weight amongst the inhibitory neurons that yields at least a 20% network performance improvement (Figure 1). The inhibition plays the role of suppressing overdriven, stereotypical firing behavior to render efficient sparse encoding of temporal information. This illustrates that the nonlinearity of a recurrent, dynamical network possesses more computational capacity than a simple feed-forward linear expansion provided by the non-connected network [1,2].

**Figure 1:**
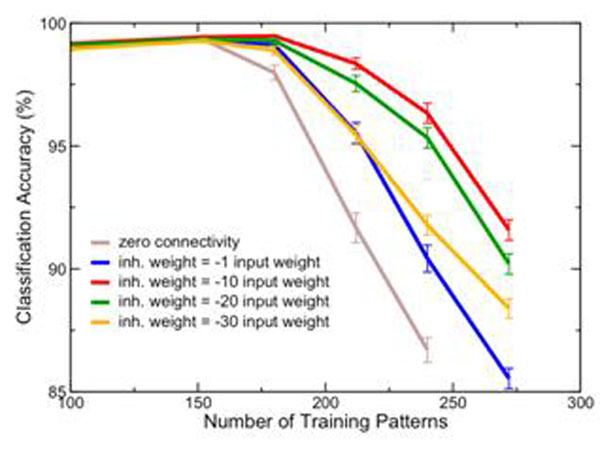
Classification accuracy vs. number of training patterns, for a fully connected inhibitory network of N = 100 neurons. Performance of the zero-connectivity network is shown as a benchmark for comparison. The N-dimensional network output is binned into n patterns, with a bin size of 30 ms, and the linear classifier is trained to separate the first n/2 patterns from the latter n/2. The input weight, with exponentially decreasing post-synaptic current (psc, with 100 ms time constant), is sub-threshold. Inhibitory network weights are reported in relation to the input weight, though the inhibitory psc time constants are much smaller (8 ms).

## References

[B1] Maass W Natschläger T Markram H Real-time computing without stable states: a new framework for neural computation based on perturbations Neural Comput 2002 14 2531 2560 10.1162/089976602760407955 12433288

[B2] Jäger H Haas H Harnessing nonlinearity: predicting chaotic systems and saving energy in wireless communication Science 2004 304 78 80 10.1126/science.1091277 15064413

